# Effects of pause-based prosodic intervention on classical Chinese poetry reading among students with reading difficulties

**DOI:** 10.3389/fpsyg.2026.1944942

**Published:** 2026-10-21

**Authors:** Yingsheng Ni, Haoyuan Zheng

**Affiliations:** 1 Suzhou Zhenhua Middle School, Suzhou, China; 2 School of Teacher Education, Guangzhou Huashang College, Guangzhou, China; 3 Department of Psychology, The Education University of Hong Kong, Hong Kong, China

**Keywords:** classical Chinese, comprehension, intervention, prosodic, reading difficulties

## Abstract

**Purpose:**

This study examined the impact of pause-based prosodic reading (PBPR) on the classical Chinese poetry (CCP) reading development of students with reading difficulties (SRD) and then compared its effects with those of meaning-based intervention (MBI).

**Methods:**

This study included 211 Grade 7 Chinese SRD who were randomly assigned to three groups: PBPR, MBI and control. Students in the PBPR group received explicit instruction in pause placement during CCP oral reading, whereas those in the MBI group received structurally equivalent instruction focusing on vocabulary, meaning-unit interpretation, imagery understanding and thematic discussion. SRD in the control group received typical CCP instruction. The intervention lasted 8 weeks, with two 45-min sessions each week. Students’ CCP comprehension, classical Chinese receptive vocabulary, reading engagement and pause-related prosodic reading were all assessed before and after the intervention.

**Results:**

Both PBPR and MBI enhanced SRD’s CCP comprehension, classical Chinese receptive vocabulary and reading engagement compared with typical instruction. Moreover, PBPR produced greater gains in boundary pause ratio and greater reductions in inappropriate pause ratio than MBI. No statistically group difference was found for mean pause duration. Furthermore, MBI improved comprehension-related outcomes but did not significantly reduce inappropriate pause ratio than typical instruction.

**Conclusion:**

The study’s findings indicate that PBPR and MBI support CCP reading development among SRD. More importantly, PBPR has unique value in improving pause-placement accuracy during CCP oral reading, suggesting that explicit pause-based scaffolding may be a useful approach for helping SRD organize compact poetic lines into meaningful oral units.

## Introduction

Pause-based prosodic reading (PBPR) plays an important role in reading comprehension because it reflects students’ ability to segment written language into meaningful syntactic and semantic units during oral reading (Rasinski et al., 2011; Rodgers et al., 2025; Wolters et al., 2022). Rather than processing text word by word, sufficient text comprehension requires students use pauses to mark phrase boundaries and organize connected language into coherent units of meaning (Dong et al., 2023; Kim et al., 2020; Wolters et al., 2022). In the present study, PBPR refers to the ability to segment written text into meaningful units through appropriate pause placement during oral reading (Kuhn et al., 2010; Rasinski et al., 2011; Rodgers et al., 2025).

Classical Chinese poetry (CCP) is a key component in Chinese language comprehension (Dong et al., 2021; Lang and Numtong, 2025; Liu et al., 2023). Unlike modern Chinese text, CCP compresses meanings into short lines, omits explicit syntactic markers and relies heavily on imagery, parallelism, rhythm and sound patterns to convey emotion and meaning (Chen et al., 2024; Dong et al., 2021; Xie and Deng, 2023). Sufficient comprehension of CCP requires students to segment compact poetic lines into meaningful units before they can construct a coherent interpretation. However, the task of CCP comprehension might be a great challenge for students with reading difficulties (SRD), who may process poetic lines word by word, pause at inappropriate points and thus fail to integrate condensed language into coherent poetic meaning (Al Otaiba et al., 2023; Dong et al., 2023; Shaaban and Mohamed, 2024).

The Implicit Prosody Hypothesis (Fodor, 2002) posits that pause placement externalizes meaning segmentation, allowing SRD to identify phrase boundaries, integrate information across words and construct coherent interpretations of text. From this perspective, PBPR might be a potential approach to enhance the ability of making meaning segmentation explicit for SRD during CCP comprehension. The PBPR intervention is expected to facilitate meaning construction by helping SRD identify phrase boundaries and organize CCP into coherent semantic units. Although previous studies have shown that meaning-based instruction (MBI) can support students’ reading comprehension, much less is known about whether explicit PBPR scaffolding can improve their CCP reading skills, particularly among SRD. Moreover, it remains unclear whether PBPR provides added value beyond a structurally equivalent MBI. Therefore, the present study examined the impact of PBPR on CCP reading development among SRD and further compared its effects with those of MBI.

## Literature review

### Classical Chinese poetry reading

In general, CCP (i.e., Tang poems) is rich in Chinese cultural heritage (Cai, 2018), and is incorporated into the Chinese language curriculum (Lang and Numtong, 2025; Liu et al., 2023). CCP includes four ‘couplets’, each comprising a pair of adjacent lines and sharing an identical number of characters, such as 5–7 words in each line (Lee et al., 2017). CCP is written in a way that represents a holistic reality that integrates the physical context with human sentiments through the expression of poetic language and the quatrain structure (Dong et al., 2024; Lee et al., 2017).

Classical Chinese poetry reading is a distinctive form of literary comprehension that requires students to construct meaning from highly condensed and structurally implicit language (Lang and Numtong, 2025; Liu et al., 2023). In the Chinese curriculum, CCP is used not only to develop literary knowledge but also to support students’ understanding of traditional culture, reading interest and language-related abilities (Ministry of Education of the People’s Republic of China, 2022). Past studies have suggested that CCP could be improved by meaning-based scaffolds across lexical interpretation, imagery construction, cultural knowledge and external learning supports (Dong et al., 2022; Liu et al., 2023; Xie and Deng, 2023). However, few studies have focused on the effects of pause, which was the typical characteristic of couplets in CCP, on students’ CCP comprehension development.

Additionally, most existing studies have focused on meaning construction after students encounter the poem, rather than on the segmentation process that occurs during oral reading. Regarding CCP comprehension, students usually need to identify how poetic lines should be grouped into meaningful units before they can infer imagery, emotion or cultural meaning. Such comprehension may begin with the students’ ability to divide compact poetic lines into meaningful phrase units. At the start of text comprehension, they need to identify where one meaningful unit ends and another begins (Dong et al., 2023; Kim et al., 2020). PBPR is directly related to this process because pause placement makes phrase-boundary segmentation observable during oral reading. Appropriate pauses may support students’ ability to organize poetic language into coherent syntactic and semantic units. Notably, this segmentation demand may be particularly challenging for SRD, who often experience problems in semantic processing, inference making, comprehension monitoring and integration across text units (Al Otaiba et al., 2023; Dong et al., 2023; Nation and Snowling, 1998). However, the effects of PBPR on SRD’s CCP comprehension remain unclear.

### Pause-based prosodic reading and reading comprehension

Prosodic reading reflects how readers organize written language into meaningful syntactic and semantic units through phrasing, pausing, intonation and expression (Kuhn et al., 2010; Rasinski et al., 2011; Wolters et al., 2022). Pause placement is the key component of prosodic reading, as pauses make phrase-boundary segmentation observable during oral reading. Insufficient pauses may reflect fragmented processing or difficulty identifying syntactic and semantic boundaries (Kuhn et al., 2010; Rasinski et al., 2011; Rodgers et al., 2025). Past studies have also suggested the pause placement and phrase-boundary pausing are two typical elements in reading prosodic, which contribute to final reading comprehension (Chung and Bidelman, 2022; Kim et al., 2021; Wolters et al., 2022). The Implicit Prosody Hypothesis (Fodor, 2002) also proposes that when students mentally project prosodic structures onto written text, these prosodic structures guide syntactic parsing and meaning construction; in this regard, pause placement represents the ability to segment written language into meaningful units.

Prior studies have reported that SRD often experience difficulties in semantic processing, inference making, comprehension monitoring and integration across textual units (Al Otaiba et al., 2023; Calet et al., 2019; Kim et al., 2021). Research on reading prosody has also reported that students’ pause placement and phrase-boundary pausing are associated with reading comprehension (Chung and Bidelman, 2022; Kim et al., 2021; Wolters et al., 2022). However, few studies have examined the effect of PBPR in SRD, and even fewer have situated this issue in structurally compact texts, such as CCP. In addition, past studies on reading comprehension intervention have suggested that intervention effects are closely related with explicit instruction from the instructor (Dong et al., 2021; Miao et al., 2026; Yuan et al., 2026), as well as students’ nonverbal intelligence (Dong et al., 2021; Kim et al., 2020; Yuan et al., 2026), working memory (Dong et al., 2023; Kim et al., 2020), prosodic reading (Rasinski et al., 2011; Rodgers et al., 2025; Wolters et al., 2022), vocabulary knowledge (Miao et al., 2026; Tong et al., 2009; Kim et al., 2020) and basic comprehension (Clemens et al., 2019; Dong et al., 2021; Kim et al., 2021), all of which should be controlled during the intervention.

### The current study

To address the aforementioned research gaps, the present study examined the effects of PBPR on CCP reading development among SRD and compared its effects with those of MBI. This study aims to address two research questions (RQs):

RQ1: Do PBPR and MBI improve CCP reading development and related abilities among SRD compared with typical instruction?

We hypothesize that students who receive PBPR or MBI would show greater improvement than those in the control group (CG) in terms of CCP comprehension, classical Chinese receptive vocabulary and reading engagement. We also expect PBPR to improve pause-related prosodic reading indicators compared with typical instruction.

RQ2: Does PBPR have different effects on CCP reading development and related abilities among SRD compared with MBI?

We hypothesize that PBPR would produce greater improvement than MBI in pause-placement accuracy, as reflected by higher boundary pause ratio and lower inappropriate pause ratio. We also hypothesize that PBPR and MBI would produce comparable improvement in CCP comprehension, classical Chinese receptive vocabulary and reading engagement.

## Methods

### Participants

The study participants were initially recruited from nine middle schools in a city in eastern China. All the participants were Grade 7 students aged 12–13 years who were identified as having reading difficulties through educational screening based on reading-comprehension performance and Chinese language teachers’ nominations. The selection criteria were as follows: (a) currently received typical education in grade 7; (b) scored at or below the 25th percentile on a grade-level Chinese reading comprehension test among the total 5,518 middle school students in the exam held in Spring 2026, relative to the full screening sample of 5,518 Grade 7 students; (c) were nominated by their Chinese language teachers as showing persistent difficulties in Chinese reading comprehension during the first semester of Grade 7, based on classroom performance and routine assessments; (d) scored at or below the 25th percentile in CCP comprehension in the Spring 2026 examination, relative to the same screening sample; (e) had no known intellectual disability, based on existing records or parent reports; and (f) agreed to participate in this study.

As a result, 240 SRD students were recruited and then randomly assigned at the individual level by drawing lots to the PBPR, MBI and control groups (80 students per group). However, 29 students dropped out during the intervention due to unforeseen reasons, such as moving to another city. Participant flow, including reasons for attrition by group, is presented in Figure 1. Attrition rates were 8.75%, 11.25 and 16.25% in the PBPR, MBI and control groups, respectively. A chi-square test indicated that attrition did not significantly differ across conditions. Finally, the PBPR group had 51 boys and 22 girls (average age = 12.52 years old, SD = 0.30), MBI had 47 boys and 24 girls (average age = 12.52 years old, SD = 0.28) and the CG had 44 boys and 23 girls (average age = 12.47 years old, SD = 0.35). Their data were used for further data analysis.

**Figure 1:**
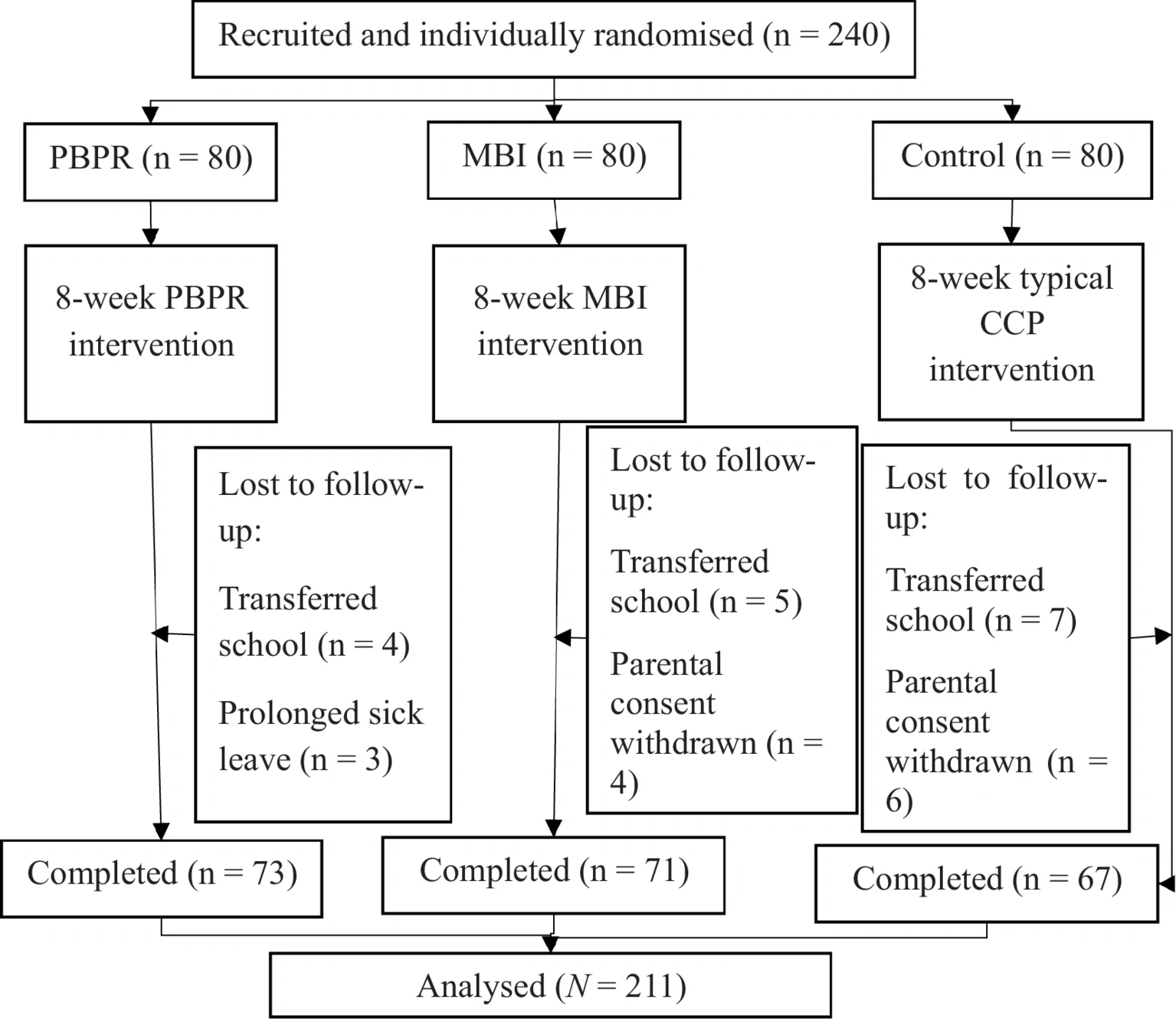
Participant flow diagram.

### Measures

#### Nonverbal intelligence

This study used Raven’s Standard Progressive Matrices to measure students’ nonverbal intelligence. This test contained 5 sections with 12 items in each section. The students were required to select one piece from 6 to 8 options to best complete the given picture. Each correct answer was given 1 point, and the maximum total score for the test was 60. The Cronbach’s α was 0.86 in the current study.

#### Working memory

This study used Zheng et al.’s (2026) Digit Span Test (developed from Han, 2011) to measure students’ working memory. In this 21-item test, the students were required to repeat a series of numbers in reverse order as presented by the instructor and clearly report their answers to the instructor. Each digit ranged from 0 to 9 and appeared only once per item. For example, if the sequence was ‘1, 4, 3, 2’, the correct response would be ‘2, 3, 4, 1’. The number of digits in each item increased with task difficulty. Each correct response was given 1 point, and the maximum score for the test was 21. The Cronbach’s α was 0.90 in the current study.

#### Chinese reading comprehension

Chinese reading comprehension was assessed for participant screening using a test used uniformly by the local education bureau for Grade 7 students in Spring 2026. The test assessed understanding of explicitly stated information and text-based inference. The test content was aligned with the Grade 7 Chinese language curriculum, and responses were scored according to a common scoring guide provided by the local education bureau. Students scoring at or below the 25th percentile relative to the full screening sample of 5,518 Grade 7 students met the general reading-comprehension screening criterion. This criterion was considered alongside teacher nominations and the CCP-comprehension criterion described in the Participants section. The test was used for educational screening and was not an intervention outcome measure or a clinical diagnostic instrument, but used as a covariable. Internal consistency was 0.81 (Cronbach’s α) in the current study.

#### Classical Chinese poetry comprehension

Classical Chinese poetry comprehension was assessed using a 10-item measure adapted from Dong et al. (2021). The adapted items and scoring key were reviewed by invited experts for their suitability for Grade 7 students and the correspondence between each poetic context and the intended target-character meaning. Students were required to translate each Classical Chinese sentence into modern Mandarin. Each item contained at least one polysemous target character whose intended meaning was constrained by the sentence context. The measure focused on students’ interpretation of contextual word meanings within poetic lines. The adaptation involved replacing the original sentences with poetic lines and modifying item wording and difficulty. Responses were scored using a predefined answer key specifying the intended contextual meaning of each target character. A response was scored as correct if it accurately conveyed that meaning. Each correct response received 1 point, yielding a maximum score of 10. Cronbach’s α was 0.83 in the current study.

#### Classical Chinese receptive vocabulary

This study used Dong et al.’s (2021) classical Chinese Receptive Vocabulary Test. In this 60-item test, the students were asked to identify the semantic meaning of a given word and then create a new double-character word from it [e.g. 爱 (like) = 爱着 (‘be like’ = like)]. The students needed to take partial knowledge of the target characters to finish the test. Each correct answer was given 1 point, and the maximum score for the test was 60. The Cronbach’s α was 0.95 in the current study.

#### Reading engagement

This study used Zhang and Xin’s (2012) Reading Engagement Scale to measure the students’ reading engagement. In this 5-item scale, the students were required to selected one description (1 = highly disagree to 5 = highly agree) from each item that best matched their current situation. A higher score indicates a higher level of reading engagement. The maximum score was 25 and the Cronbach’s α was 0.91.

#### Prosodic reading

In this study, we used an adapted version of Chung and Bidelman’s (2022) acoustic approaches to oral reading prosody test by replacing the Mandarin sentence context using the CCP. The test was driven by E-prime 3.0. The students’ voices were recorded during the test, and we only selected measures that focused on the pause placement and phrase-boundary pausing in reading. Then, the students were asked to read aloud three unfamiliar CCP individually in a quiet room. Each test form contained a total of 12 poetic lines comprising 84 Chinese characters. The poems were not used during the intervention and were matched across testing points in terms of poetic form, number of characters, vocabulary difficulty, syntactic complexity and familiarity.

Before coding, a pause-boundary key was developed for each poem. Five experts in Chinese language education and CCP teaching, all of whom were senior lecturers, independently identified acceptable pause boundaries on the basis of syntactic structure, semantic units, poetic line structure and meaning integration. The experts then compared their judgments and discussed any disagreements with reference to these criteria until unanimous agreement was reached. Only boundaries agreed upon by all five experts were included in the final pause-boundary key. Each assessment form included 24 target pause boundaries, corresponding to two internal target pause boundaries per poetic line.

Next, the students’ oral readings were digitally recorded and analyzed using Praat. Here, a *silent pause* was defined as an acoustic silence of at least 250 ms. Acoustic coding focused on within-line silent pauses. Line-final pauses, intervals between poems, and silence before and after the reading were excluded. A valid silent pause was defined as a silent interval lasting at least 250 ms between spoken segments within a poetic line. Intervals shorter than 250 ms were not included in the pause measures. Pauses exceeding 3,500 ms were manually inspected: those attributable to the reading itself, including reading-related hesitation, were retained, whereas intervals attributable to recording interruptions, coughing, or other identifiable nonreading events were excluded. The same inclusion criteria were applied to all three acoustic indicators.

Recordings were analyzed in Praat using manual annotation. Coders inspected the waveform and spectrogram and listened to the recordings to locate within-line silent pauses. Pause onset was marked at the end of the preceding speech sound, and pause offset at the beginning of the following speech sound. Consonant closures were distinguished from pauses through acoustic inspection and auditory checking. Audible breathing was excluded from the measured silent interval, and silent intervals separated by an audible breath were evaluated separately against the 250-ms inclusion threshold.

The final threshold was determined based on prior pause-duration research and pilot inspection of the recordings. The acoustic indicators included (a) mean pause duration, (b) boundary pause ratio and (c) inappropriate pause ratio. Three acoustic indicators were calculated separately for each student at pretest and posttest by pooling the coded pauses across the three assessment poems. Boundary pause ratio was calculated as the number of target boundaries at which a valid pause occurred (H) divided by the total number of target boundaries (*T* = 24). Each target boundary contributed at most one count to H (Boundary pause ratio = H / 24).

Inappropriate pause ratio was calculated as the number of valid pauses at inappropriate within-line locations (U) divided by the total number of valid within-line pauses (N). Locations were classified using the final expert-agreed boundary key. All acceptable within-line pause locations identified by the expert panel were included in the 24 target boundaries; pauses at other within-line locations were classified as inappropriate (Inappropriate pause ratio = U/N). Mean pause duration was calculated as the summed duration of valid pauses at target boundaries divided by the number of these pauses (A), where (d_j_) denotes the duration of the (j)_th_ pause in milliseconds. Higher boundary pause ratios indicated that more target boundaries were realized with pauses, whereas higher inappropriate pause ratios indicated a greater proportion of pauses at inappropriate locations. Mean pause duration described pause timing at target boundaries. No cases with (*N* = 0) or (*A* = 0) occurred at either pretest or posttest.

We used the raw acoustic indicators of boundary pause ratio, inappropriate pause ratio and mean pause duration at appropriate boundaries. Boundary pause ratio ranged from 0 to 1, with higher values indicating more frequent pausing at expert-identified phrase boundaries. Inappropriate pause ratio also ranged from 0 to 1, with lower values indicating fewer pauses at nontarget locations. A randomly selected 30% of recordings were independently double-coded by two trained coders who were blind to the students’ group assignment and testing times. Intercoder reliability regarding mean pause duration, boundary pause ratio and inappropriate pause ratio was assessed before disagreements were resolved, with ICCs ranging from 0.81 to 0.92. Agreement for categorical pause-boundary coding was also high at κ = 0.92. Disagreements were resolved through discussions before final scoring.

### Research procedure

This study received ethnical approval from the Institutional Review Board of the designated universities. We collected the students’ oral assent and their parents’ formal consent before initiating the study. This study recruited 30 research assistants (RAs) who had successful experience in administering psychological and language measures to students.

For the pretest, all the students received individual tests inside one silent classroom. These tests assessed their nonverbal intelligence, working memory, classical Chinese poetry comprehension, classical Chinese receptive vocabulary, reading engagement and prosodic reading. Chinese reading-comprehension scores were obtained from the Spring 2026 screening assessment. The students took around 2 h to complete all the tests under supervision of the RAs.

Prior to the interventions, we trained these 30 RAs with the necessary techniques they needed to use during the intervention implementation to these. The intervention lasted 8 weeks, with two sessions per week and 45 min per session. Across the 16 sessions, the students in all three conditions received the same poems in the same order. The detailed 8-week session schedule, sample lesson scripts and student worksheets are presented in Appendix I.

To ensure that the intervention proceeded as expected, weekly supervision meetings were held to review implementation quality, discuss difficulties encountered during instruction and prevent deviation from the assigned condition. The RAs also completed a session log after each lesson in which they recorded the poem taught, the activities completed, student attendance, feedback provided, deviations from the lesson script and notes of unexpected events. The RAs were also required to make audio recordings during each intervention session and submit them to the research team before the next workday.

We administered the posttest immediately after the intervention when the students completed the last session. The posttest assessed the same outcome domains as the pretest: classical Chinese poetry comprehension, classical Chinese receptive vocabulary, reading engagement and prosodic reading. Neither the pretest nor the posttest materials included any of the 16 poems taught during the intervention. For prosodic reading, different sets of poems, matched on the characteristics described in the Prosodic Reading section, were used at pretest and posttest. For classical Chinese poetry comprehension and classical Chinese receptive vocabulary, identical assessment items were administered at pretest and posttest in all three groups; no alternate forms were used.

### Intervention materials

Initially, we used the following criteria to select potential poems: (a) had a clear seven-character line structure and imagery words suitable for Grade 7 students (Dong et al., 2024); (b) shared similar reading difficulties across poetic form, vocabulary difficulty, syntactic complexity, imagery density and curriculum relevance, which we evaluated using Jin and Lu’s (2018) database; and (c) contained identifiable meaning units and phrase boundaries that allowed the students to practice pause-boundary marking during oral reading. Poems that were likely to be highly familiar to students or had been recently taught in regular Chinese language classes were excluded. As a result, we obtained a total of 19 poems.

The same five experts evaluated the appropriateness of the 19 candidate poems against the selection criteria described above. Any disagreements were discussed with reference to these criteria until unanimous agreement was reached. Only poems approved by all five experts were included, yielding 16 poems for the intervention. As a result, the intervention materials consisted of 16 seven-character Classical Chinese poems (Appendix II). Only one poem was given during each intervention and every poem was only learned once during the intervention.

### Research assistant training

The research team conducted two-hour workshops for the RAs assigned to the three groups (PBPR, MBI and control) RAs. During the workshop, the research team members introduced the theoretical rationale of the study, the characteristics of SRD, lesson scripts, allowed and prohibited feedback, classroom management, fidelity procedures, practice teaching and the necessary techniques to be used the PBPR, MBI and control groups. Each RA only underwent one corresponding workshop (PBPR, MBI and control) for the intervention they would implement for 8 weeks.

After the workshop, the research team members played the role of SRD, and the RA used the given poem item [送元二使安西] (‘Sending Yuan Er on a Mission to Anxi’). Only those RAs who mastered the necessary intervention techniques were considered qualified educators. For example, the PBPR group RAs who successfully performed identifying and teaching meaning-based pause boundaries, guiding students to mark pauses, conducting marked and faded oral reading and providing pause-focused feedback were regarded as qualified educators. MBI group RAs who successfully performed explaining vocabulary, guiding meaning-unit interpretation, discussing imagery and emotion and providing meaning-focused feedback without explicit pause instruction were considered qualified educators. The CG RAs who successfully performed conventional poetry instruction, including regular oral reading, vocabulary explanation, sentence translation, brief thematic explanation, recitation practice and comprehension exercises, without systematic PBPR or structured MBI prompts, were regarded as qualified educators.

We evaluated each RA performance by using a condition-specific implementation checklist (Appendix III), which included adherence to the lesson script, correct usage of the assigned instructional techniques, appropriate feedback, avoidance of prohibited techniques, classroom management and time control. All 30 RAs passed the practice items in the first round.

### Pause-based prosodic reading group

The students in the PBPR group received explicit instruction in pause placement during the oral reading of the CCP. The RAs’ prompts focused on pause placement and phrase-boundary pausing, without explicit instructions related to stress, intonation, emotional expression, musical rhythm or performance-based recitation. Each session included brief text access, pause-boundary modelling, student pause marking, guided oral reading and pause-focused reflection.

First, the RA explained the necessary pronunciations and difficult words, ensuring that SRD could read the poem aloud and was not used as systematic vocabulary instruction, imagery analysis, thematic interpretation or sentence-level explanation. Second, the RA used an example to elaborate on the process of identifying meaningful pause boundaries based on semantic units and syntactic relations. Third, SRD marked pause boundaries in the poem using slashes. Fourth, the students practiced reading the poem aloud with full pause marks, with partially faded pause marks and finally without pause marks. Fifth, they completed a short reflection task explaining why particular pauses helped them understand the poem.

The RAs in this condition provided feedback only on pause-related features, such as whether SRD paused at meaningful boundaries, they avoided pausing within words or phrases, their reading was overly fragmented and pause placement helped organise the poem into coherent meaning units. Notably, the RAs in the PBPR group were not allowed to provide feedback on stress, intonation, emotional expression or performance quality.

### Meaning-based intervention group

Students in the MBI group received structurally equivalent instruction, with a focus on meaning construction in CCP. The MBI condition was matched with the PBPR condition in terms of text materials, instructional time, RA contact, oral reading opportunities, peer discussion, written reflections and feedback frequency. However, the MBI prompts focused on semantic meaning identification rather than prosodic reading. Each session included vocabulary explanation, meaning-unit interpretation, imagery analysis, guided reading, peer discussion and written reflection.

The RA introduced the poem, explained difficult vocabulary words, guided SRD to interpret meaning units, helped them identify key imagery and emotional words and support literal and inferential understanding of the poem. SRD were also required to read the poem aloud, discuss the poem with their peers and complete a written reflection task. However, the RAs in the MBI group were not allowed to ask students to mark pause boundaries, teach the students where to pause and provide pause-focused feedback. Regarding the interactive feedback during intervention, the RAs in this group focused on vocabulary meaning, sentence interpretation, imagery understanding, thematic interpretation and accuracy of explanation.

### Control group

SRD in the CG received typical CCP instruction, which was the same as school CCP instruction, but using the same learning materials as the PBPR and MBI groups. Each session, which lasted for 45 min and held twice a week, included typical oral reading, vocabulary explanation, sentence translation, brief background explanation, recitation practice and comprehension exercises. Unlike the PBPR condition, the CG condition did not include systematic pause-boundary marking, faded pause reading, pause-focused feedback or reflection on pause placement. The CG also did not include structured MBI prompts or systematic meaning-unit scaffolding.

### Data analysis

To ensure the baseline of SRD across PBPR, MBI and control groups, we used Chi-square tests and one-way ANOVAs to examine whether the three groups differed in demographic characteristics and pretest measures before the intervention. Baseline demographic characteristics and pretest scores were also compared between completers and non-completers using independent-samples t-tests for continuous variables and a chi-square test for gender. At posttest, repeated-measures ANCOVAs were conducted for each outcome, with time (pretest and posttest) as the within-subject factor and condition (PBPR, MBI and control) as the between-subject factor, controlling for age, gender, nonverbal intelligence, working memory and Chinese reading comprehension. These analyses examined whether changes from pretest to posttest differed across conditions. Residual normality, homogeneity of variance and homogeneity of regression slopes were examined. The checks indicated no substantial violations of these assumptions. Furthermore, the effect sizes were measured using partial eta-squared (partial *η*^2^), with partial *η*^2^ = 0.01, 0.06 and 0.14 indicating small, medium and large effect sizes, respectively, following the rule of thumb for effect sizes (Cohen, 1988). Additionally, Bonferroni correction was applied to pairwise comparisons between conditions at posttest within each outcome. Analyses included the 211 students with complete pretest and posttest data, missing data were not imputed.

### Fidelity of implementation

The research team transcribed the audio recordings and conducted fidelity checks to determine the following: (1) the length of each intervention session as well as the frequencies of (2) of pause-based prompts during poetry reading, (3) meaning-based prompts during poetry reading and (4) the use of other instructional techniques in each session.

We also randomly selected 30% of the intervention recordings that were independently coded by a second trained coder, who was blind to the study hypotheses and group assignment. Intercoder reliability was acceptable for session duration, pause-based prompts, meaning-based prompts and other instructional techniques, with ICCs ranging from 0.81 to 0.92. Disagreements were resolved through discussions before final coding.

## Results

### Descriptive analysis

Table 1 presents the pretest and posttest means and standard deviations for the three intervention groups. The results of the ANOVA (Table 2) and chi-square tests showed that the three groups (PBPR, MBI and control) did not significantly differ in terms of demographical factors (*p*s > 0.10), indicating a similar demographical baseline and CCP-related abilities across these groups. In addition, z scores showed that all data were within ±2, revealing the absence of outliers in this study.

**Table 1:** Descriptive information of students with reading difficulties across groups.

Variable		Pretest		Posttest	
		Mean	SD	Mean	SD
PBPR	Raven	28.67	3.99		
	WM	10.86	2.41		
	MCRC	6.15	1.78		
	CCPC	2.81	1.44	4.89	1.51
	CCRV	29.10	7.00	32.88	7.21
	RE	13.63	2.65	15.78	3.08
	PR_MPD	921.26	187.80	855.77	125.11
	PR_BPR	0.37	0.11	0.66	0.13
	PR_IPR	0.42	0.10	0.22	0.12
MBI	Raven	29.24	3.61		
	WM	11.37	2.72		
	MCRC	6.21	1.68		
	CCPC	3.04	1.47	4.63	1.54
	CCRV	29.48	6.65	33.23	6.81
	RE	13.93	3.49	15.16	3.48
	PR_MPD	944.96	235.17	894.48	185.34
	PR_BPR	0.39	0.12	0.47	0.15
	PR_IPR	0.44	0.09	0.37	0.10
Control	Raven	28.82	3.78		
	WM	10.72	2.61		
	MCRC	5.85	1.81		
	CCPC	2.90	1.38	3.64	1.50
	CCRV	28.28	5.31	29.52	5.74
	RE	13.39	2.45	13.97	2.87
	PR_MPD	961.00	231.83	951.90	178.37
	PR_BPR	0.38	0.11	0.41	0.12
	PR_IPR	0.41	0.10	0.38	0.12

WM = working memory, MCRC = Chinese reading comprehension, CCPC = classical Chinese poetry comprehension, CCRV = classical Chinese receptive vocabulary, RE = reading engagement, PR_MPD = prosodic reading mean pause duration, PR_BPR = prosodic reading boundary pause ratio, PR_IPR = prosodic reading inappropriate pause ratio.

**Table 2:** Baseline comparison.

Variable	*F*	*p*	Partial *η*^2^	Comparison
Raven	0.43	0.65	0.004	PBPR = MBI=Control
WM	1.22	0.30	0.012	BPR = MBI=Control
MCRC	0.83	0.44	0.008	BPR = MBI=Control
CCPC	0.49	0.61	0.005	BPR = MBI=Control
CCRV	0.63	0.54	0.006	BPR = MBI=Control
RE	0.60	0.55	0.006	BPR = MBI=Control
PR_MPD	0.59	0.56	0.006	BPR = MBI=Control
PR_BPR	0.33	0.72	0.003	BPR = MBI=Control
PR_IPR	1.14	0.32	0.011	BPR = MBI=Control

CCPC = classical Chinese poetry comprehension, CCRV = classical Chinese receptive vocabulary, RE = reading engagement, PR_MPD = prosodic reading mean pause duration, PR_BPR = prosodic reading boundary pause ratio, PR_IPR = prosodic reading inappropriate pause ratio.

Research question 1: do PBPR and MBI improve CCP reading development and related abilities among SRD compared with typical instruction?

In the repeated ANCOVA (Table 3), we set SRD’s age, gender, nonverbal intelligence, working memory and Chinese reading comprehension as the covariables. Group conditions (PBPR, MBI and control) were set as between-subject factors and time was set as within-subject variables. SRD’s CCP comprehension, classical Chinese receptive vocabulary, reading engagement, mean pause duration, boundary pause ratio and inappropriate pause ratio were all set as dependent variables. The results indicated that the time effects were significant across all selected measures (*p*s. < 0.001, *η*^2^ range: 0.063–0.806). The time × group (PBPR, MBI and control) was not significant in mean pause duration (*p* = 0.11, *η*^2^ = 0.021), but the time × group (PBPR, MBI and control) were significant in other selected measures (*p*s. < 0.001, *η*^2^ range: 0.100–0.743). The values of effect size *η*^2^ ranged from medium to large in terms of the time effect and the interaction effect between group conditions and time. This result indicated that SRD in the PBPR, MBI and control groups demonstrated differences in their performances between the pre- and posttests.

**Table 3:** Group comparison.

Variable	Time	Partial *η*^2^	Time × Condition	Partial *η*^2^	Comparison
CCPC	637.08^***^	0.754	44.14^***^	0.298	PBPR = MBI > Control
CCRV	319.94^***^	0.606	24.90^***^	0.193	PBPR = MBI > Control
RE	96.58^***^	0.317	11.51^***^	0.100	PBPR = MBI > Control
PR_MPD	13.92^***^	0.063	2.24	0.021	PBPR = MBI=Control
PR_BPR	865.66^***^	0.806	301.12^***^	0.743	PBPR>MBI > Control
PR_IPR	636.94^***^	0.754	181.05^***^	0.635	PBPR<MBI=Control

^***^p < 0.001; CCPC = classical Chinese poetry comprehension, CCRV = classical Chinese receptive vocabulary, RE = reading engagement, PR_MPD = prosodic reading mean pause duration, PR_BPR = prosodic reading boundary pause ratio, PR_IPR = prosodic reading inappropriate pause ratio.

Given the significant time × group (PBPR, MBI and control) interaction effects, except the mean pause duration, we conducted simple effect analysis for other selected variables. The results showed that at posttest, the MBI group had similar inappropriate pause ratio development with the CG (mean difference = 0.02, *p* = 0.76). PBPR and MBI had better performances than the CG across the other variables (*p*s < 0.05).

Research question 2: does PBPR have different effects on CCP reading and related abilities among SRD compared with MBI?

Further simple effect analysis revealed that the PBPR group had higher boundary pause ratio (mean difference = 0.19, *p* < 0.001) and lower inappropriate pause ratio (mean difference = 0.15, *p* < 0.001) development than the MBI group. However, the PBPR group had similar development with the MBI group in terms of CCP comprehension (mean difference = 0.26, *p* = 0.68), classical Chinese receptive vocabulary (mean difference = 1.35, *p* = 0.53) and classical Chinese reading engagement (mean difference = 0.63, *p* = 0.55).

### Fidelity of implementation

Table 4 presents the one-way ANOVA results, revealing that instructors in all three groups maintained consistent duration for each intervention session [*F* = 1.06, *p* = 0.35, *η*^2^ = 0.010]. Meanwhile, significant group differences were found in the frequencies of pause-based prompts during classical Chinese poetry reading [*F* = 396.72, *p* < 0.001, *η*^2^ = 0.792], meaning-based prompts [*F* = 342.15, *p* < 0.001, *η*^2^ = 0.765] and adoption of other instructional techniques in each session [*F* = 22.89, *p* < 0.001, *η*^2^ = 0.180]. Further *post hoc* test results suggested that the RAs in the PBPR group used pause-based prompts far more frequently than those in the MBI (mean difference = 8.35, *p* < 0.001) and control groups (mean difference = 8.41, *p* < 0.001). No significant difference was observed in the use of pause-based prompts between the MBI and control groups (mean difference = 0.06, *p* = 0.71).

**Table 4:** Fidelity of intervention implementation.

Coding indicators	PBPR		MBI		Control	
	*Mean*	*SD*	*Mean*	*SD*	*Mean*	*SD*
Duration for each intervention session (unit: minutes)	44.92	0.78	45.05	0.81	44.87	0.75
Quantity of pause-based prompts per intervention session	8.76	2.94	0.41	0.63	0.35	0.58
Quantity of meaning-based prompts per intervention session	0.38	0.59	7.92	2.81	0.44	0.65
Quantity of other instructional techniques in each intervention session	0.52	0.61	1.18	0.97	1.24	1.02

In terms of meaning-based prompts, the RAs in the MBI group applied this type of prompt significantly more often than the RAs in the PBPR group (mean difference = 7.54, *p* < 0.001) and the control group (mean difference = 7.48, *p* < 0.001). The frequency of meaning-based prompts used by RAs in the PBPR group was comparable to that in the CG (mean difference = 0.06, *p* = 0.68). For other instructional techniques, *post hoc* comparisons indicated that the CG more frequently used other instructional techniques than the PBPR group (mean difference = 0.72, *p* < 0.001). The MBI group also adopted other techniques more frequently than the PBPR group (mean difference = 0.66, *p* < 0.001), while there was no statistical difference between the MBI and control groups (mean difference = 0.06, *p* = 0.73). The results indicated that the RAs in the PBPR and MBI groups implemented the corresponding intervention strategies as intended, in line with the research design. In comparison, the RAs in the CG carried out conventional classical Chinese poetry teaching without systematic pause-based prompts or structured MBI prompts.

## Discussion

This study examined the effects of PBPR intervention on CCP reading among Chinese SRD. We obtained two main findings. First, both PBPR and MBI improved SRD’s CCP comprehension, classical Chinese receptive vocabulary and reading engagement. Second, PBPR showed a unique advantage over MBI in pause-related prosodic reading indicators in terms of greater improvement boundary pause ratio and greater reduction in inappropriate pause ratio. However, PBPR did not produce greater improvement than MBI in mean pause duration.

### Effects of PBPR and MBI on students’ CCP reading development

The present findings are in line with previous studies, which showed that explicit reading scaffolds can support students’ reading comprehension, vocabulary learning and reading-related affective development (Dong et al., 2022; Liu et al., 2023; Rasinski et al., 2011; Rodgers et al., 2025; Wolters et al., 2022; Xie and Deng, 2023). In the current study, both PBPR and MBI led to greater gains than typical education in CCP comprehension, classical Chinese receptive vocabulary and reading engagement.

In the current study, both PBPR and MBI provided SRD with more explicit access to the internal structure of CCP than typical education. For SRD, the main characteristics of CCP (e.g., condensed language, implicit syntactic relations and highly compact semantic units) may increase the difficulty of identifying how poetic lines should be organized into meaningful units. The PBPR group received instruction of marking phrase boundaries, read poems with guided pauses and reflect on how pause placement supported meaning construction. Although the PBPR intervention did not provide systematic meaning-based explanation, pause-boundary instruction may have helped the students organise poetic lines into more coherent syntactic and semantic units, thereby supporting CCP comprehension. Meanwhile, the MBI group received structured instruction in vocabulary meaning, meaning-unit interpretation, imagery analysis, thematic understanding, peer discussion and written reflection, which directly supported the lexical and semantic processes required for CCP comprehension.

An alternative reason for the current finding is the provision of structured and supportive instructions in both interventions. Compared with typical education, the PBPR and MBI conditions provided clearer learning goals, more guided practice and more condition-specific feedback. For SRD, such support may reduce the frustration commonly associated with CCP reading and increase their sense of control during poem learning (Xu et al., 2024). In PBPR, SRD visibly marked and practiced pause boundaries, while in MBI, they received direct support in interpreting vocabulary, imagery and meaning. These concrete learning support may have made CCP reading more accessible, contributing to higher reading engagement after the intervention. This finding suggests that SRD can benefit from structured support when learning CCP, regardless of whether the support primarily targets pause-based segmentation or meaning construction.

### Added value of PBPR over MBI

This finding is consistent with the proposed role of prosodic segmentation in syntactic parsing and meaning construction during reading (Fodor, 2002). This finding is partially consistent with prior research suggesting that pause placement and phrase-boundary pausing are important indicators of how readers organize written language into meaningful syntactic and semantic units during oral reading (Chung and Bidelman, 2022; Kim et al., 2021; Rasinski et al., 2011; Rodgers et al., 2025; Wolters et al., 2022). Similarly, the present study showed that pause-related prosodic reading can be directly improved among Chinese SRD through explicit pause-based scaffolding in the context of CCP.

In the current study, PBPR directly targeted the mapping between written poetic lines and meaningful oral units. In the PBPR condition, SRD were guided to identify phrase boundaries, mark pauses with visual symbols, practice oral reading with full and faded pause marks and receive feedback on whether their pauses occurred at meaningful boundaries. As such, PBPR’s instructional procedures may have helped SRD apply the taught pause-placement strategies when reading CCP lines. By contrast, MBI focused on vocabulary explanation, meaning-unit interpretation, imagery analysis, thematic understanding, peer discussion and written reflection. These activities directly supported SRD’s semantic understanding of CCP, which may explain why the students in the PBPR and MBI groups showed similar development in CCP comprehension, classical Chinese receptive vocabulary and reading engagement.

However, MBI did not require the students to mark pause boundaries, practice pause-based oral reading or receive pause-focused corrective feedback. Therefore, although MBI helped the students understand poetic meaning, it may not have provided sufficient procedural practice for them to externalize meaning segmentation through accurate pause placement during oral reading. PBPR’s advantage over MBI in boundary pause ratio and inappropriate pause ratio may partly reflect the direct alignment between explicit pause-placement training and these outcome measures. Because the assessment poems were not taught during the intervention, the findings also suggest that students applied the trained pause-placement skills to untrained poems.

Another interesting finding is similar development of mean pause duration between PBPR and MBI. In the current study, PBPR was designed to teach students where to pause and where not to pause, rather than how long each pause should last. As a result, the PBPR and MBI interventions provided similar oral reading opportunities and general instructional support. Hence, the students in the PBPR and MBI groups may have developed comparable temporal pause length. Thus, the similar development of mean pause duration does not weaken the added value of PBPR. Such a result might also indicate that PBPR primarily improved the structural accuracy of pause placement rather than the temporal duration of pauses.

### Limitations, implications and future directions

This study has several limitations that should be addressed in future studies. First, all the participants were Chinese SRD in Grade 7. Past studies have shown that learners with different reading abilities (e.g., poor comprehenders and poor decoders) and psychological characteristics had various learning outcomes across cognitive and affective factors (Dong et al., 2023; Miao et al., 2025; Xu et al., 2024). This may limit the generalizability of the findings to students from other grades, regions, school systems or reading profiles. Hence, future studies should replicate the present design with students from different grade levels, regions and school contexts. It would also be useful to examine whether PBPR has similar effects among typical readers, younger students, older students and SRD with different reading difficulty profiles. In addition, school- and class-level clustering was not accounted for in the analyses, which may have affected the precision of the estimated intervention effects.

Second, the posttest was administered immediately after the intervention, and no delayed posttest or follow-up assessment was conducted. The findings therefore demonstrate immediate intervention effects, while the maintenance of these effects over time remains unknown. Future research should include delayed posttests, such as 1-month, 3-month or 6-month follow-up assessments, to examine whether gains in CCP comprehension, classical Chinese receptive vocabulary, reading engagement and pause-related prosodic reading are sustained after the intervention ends. Third, the intervention was implemented by trained RAs under relatively controlled conditions. While this design ensured internal validity and treatment fidelity, it may not fully represent how PBPR would be delivered by regular Chinese language teachers in ordinary classrooms. Therefore, future studies should examine the feasibility and effectiveness of teacher-delivered PBPR in regular school settings. Fourth, identical items were used at pretest and posttest to assess CCP comprehension and classical Chinese receptive vocabulary. Repeated exposure may have increased familiarity with the assessed materials, such that observed gains may partly reflect practice or memory effects. Although all three groups completed the same assessments, allowing between-group comparisons to account partly for shared testing effects, familiarity may have interacted differently with instruction across conditions. The intervention effects on these outcomes should therefore be interpreted cautiously, and transfer to previously unencountered assessment items remains uncertain. This concern applies to the comprehension and vocabulary assessments, which reused the same items at pretest and posttest, despite not including the intervention poems. The prosodic reading assessment used different poems at the two testing occasions, none of which were taught during the intervention. Future studies should use parallel assessment forms with comparable difficulty to reduce this concern and examine whether gains extend to new materials. Finally, the pause-placement measures were closely aligned with the skills explicitly taught in PBPR. The observed gains therefore provide the clearest evidence for improvement in the targeted oral-reading skills. Future research could incorporate independent measures of syntactic parsing, semantic integration, and working memory to examine the cognitive processes underlying these instructional effects.

The present study also has several theoretical and practical implications. First, it extends the literature on prosodic reading by situating pause-based prosodic segmentation in the context of CCP reading among Chinese SRD. The present findings highlight pause placement as a teachable aspect of oral reading and show that students can apply the trained skills to untrained CCP materials. Second, the current findings suggest that explicit instruction in pause placement can make this otherwise implicit segmentation process more visible and teachable for SRD. PBPR makes phrase-boundary segmentation explicit through pause-placement practice. A possible explanation for its instructional benefits is that this practice helps Chinese SRD attend to syntactic and semantic groupings within CCP lines. This may be especially valuable when reading structurally compact texts such as CCP.

Regarding the practical implications, first, the PBPR can be used as a feasible instructional strategy for supporting SRD in CCP reading. In particular, teachers may guide students to identify meaning-based pause boundaries, mark pauses with visual symbols, practice oral reading with full and faded pause marks and receive feedback on whether their pauses occur at appropriate boundaries. These procedures can make the structure of compact poetic lines more visible, thus helping SRD read CCP by meaningful phrase units rather than isolated words.

Second, PBPR should not replace meaning-based instruction. The current findings indicated that MBI was effective in supporting CCP comprehension, receptive vocabulary and reading engagement, whereas PBPR showed added value mainly in pause-related prosodic reading. Therefore, teachers and instructional designers may combine the two approaches according to instructional goals. Meaning-based scaffolding can be used to support vocabulary explanation, imagery understanding and thematic interpretation, while PBPR can be used when students have difficulty segmenting poetic lines or produce inappropriate pauses during oral reading.

## Conclusion

This study provided evidence indicating that PBPR scaffolding can support CCP reading among Chinese SRD. More importantly, PBPR produced a distinctive advantage over MBI in boundary pause ratio and inappropriate pause ratio, indicating that explicit pause instruction can improve students’ pause-placement accuracy during CCP oral reading. The findings also extend existing research on reading prosody by showing that students can apply explicitly taught pause-placement skills to CCP materials not used during instruction. These results suggest that explicit pause-based scaffolding offers a targeted instructional approach for supporting pause placement among Chinese SRD when reading CCP.

## Data Availability

The data that support the findings of this study are available from the corresponding author upon reasonable request.
