# Supplementary material for: Effects of pause-based prosodic intervention on classical Chinese poetry reading among students with reading difficulties
Source: Front Psychol. 2026 Oct 21;17:1944942. doi: 10.3389/fpsyg.2026.1944942 (PMC13731394; doi:10.3389/fpsyg.2026.1944942)
Supplement: Supplementary file 1 [file Supplementary_file_1.docx]

Appendix I

The intervention lasted eight weeks, with two 45-min sessions per week. Across the 16 sessions, students in the three conditions studied the same set of seven-character Classical Chinese poems in the same order. The poems used during the intervention were not used in the pretest and posttest.

**Table A1**

*Overview of the Eight-Week Intervention Schedule*

| Week | Session | Poem Type | Pause-Based Prosodic Scaffolding Group | Meaning-Based Active Control Group | Standard-Practice Control Group |
| --- | --- | --- | --- | --- | --- |
| 1 | 1 | Seven-character poem | Introduce meaningful pause placement; distinguish phrase-based reading from word-by-word reading | Introduce keyword identification and basic meaning explanation | Regular oral reading, vocabulary explanation, and sentence translation |
| 1 | 2 | Seven-character poem | Model how to identify pause boundaries based on meaning units | Guide students to identify key words and explain literal meanings | Regular oral reading, sentence translation, and recitation |
| 2 | 3 | Seven-character poem | Practice marking pause boundaries in two-part line structures | Practice identifying imagery words and sentence meaning | Vocabulary explanation, translation, and comprehension questions |
| 2 | 4 | Seven-character poem | Practice avoiding inappropriate pauses within words or meaning units | Practice explaining imagery and emotional meaning | Regular reading, recitation, and textbook-style exercises |
| 3 | 5 | Seven-character poem | Practice pause placement in lines with parallel structures | Practice explaining parallel images and their meanings | Background explanation, sentence translation, and recitation |
| 3 | 6 | Seven-character poem | Practice guided oral reading with full pause marks | Practice guided oral reading with meaning-focused discussion | Regular oral reading and comprehension exercises |
| 4 | 7 | Seven-character poem | Practice guided oral reading with partially faded pause marks | Practice interpreting meaning units with reduced prompts | Regular explanation, translation, and recitation |
| 4 | 8 | Seven-character poem | Practice explaining why particular pauses clarify meaning | Practice explaining why keywords or images clarify meaning | Regular review and comprehension exercises |
| 5 | 9 | Seven-character poem | Students independently mark pause boundaries with RA feedback | Students independently identify key words and imagery with RA feedback | Regular reading, translation, and recitation |
| 5 | 10 | Seven-character poem | Students revise inappropriate pause marks and reread the poem | Students revise inaccurate meaning explanations and reread the poem | Regular comprehension questions and review |
| 6 | 11 | Seven-character poem | Practice independent oral reading without pause marks | Practice independent meaning explanation without structured prompts | Regular oral reading and textbook-style exercises |
| 6 | 12 | Seven-character poem | Apply pause-boundary strategy to a new poem | Apply meaning-based interpretation strategy to a new poem | Regular explanation, translation, and recitation |
| 7 | 13 | Seven-character poem | Transfer practice: identify pause boundaries in an unfamiliar poem | Transfer practice: identify key meanings in an unfamiliar poem | Regular transfer exercises |
| 7 | 14 | Seven-character poem | Transfer practice: independent oral reading and pause reflection | Transfer practice: independent interpretation and meaning reflection | Regular review and comprehension practice |
| 8 | 15 | Seven-character poem | Consolidation: faded scaffolding and independent reading | Consolidation: reduced meaning prompts and independent explanation | Regular review and recitation |
| 8 | 16 | Seven-character poem | Final consolidation: independent pause-based oral reading | Final consolidation: independent meaning-based explanation | Final regular review and comprehension exercises |

Table A2

*Standard 45-Min Session Structure Across Conditions*

| Time | Pause-Based Prosodic Scaffolding Group | Meaning-Based Active Control Group | Standard-Practice Control Group |
| --- | --- | --- | --- |
| 5 min | Brief pronunciation and difficult-word access | Brief pronunciation and difficult-word access | Brief pronunciation and difficult-word access |
| 10 min | RA models how to identify meaning-based pause boundaries | RA explains key words, imagery, or sentence meaning | RA provides conventional sentence translation or background explanation |
| 10 min | Students mark pause boundaries using slashes | Students mark key words, imagery words, or meaning units | Students complete regular vocabulary or translation exercises |
| 12 min | Guided oral reading: full pause marks, faded pause marks, independent reading | Guided oral reading with meaning-focused discussion but no pause feedback | Regular oral reading, recitation, or textbook-style reading practice |
| 5 min | Students explain why selected pauses help meaning construction | Students explain how selected words or images help meaning construction | Students answer regular comprehension questions |
| 3 min | Exit check on pause-boundary understanding | Exit check on meaning understanding | Exit check on literal meaning or translation |

**Treatment Differentiation Rules**

In the PBPR group, research assistants provided explicit feedback on whether students pause at meaningful boundaries, avoid pausing within words or meaning units, reduce word-by-word reading, and organize poetic lines into coherent phrase units. They should not provide explicit feedback on stress, intonation, emotional expression, musical rhythm, or performance quality.

In the MBI group, research assistants provided feedback on vocabulary meaning, sentence interpretation, imagery understanding, thematic interpretation, and accuracy of explanation. They should not ask students to mark pause boundaries, teach where to pause, correct pause placement, or provide pause-focused feedback.

In the control group, research assistants facilitated conventional CCP instruction, including regular oral reading, vocabulary explanation, sentence translation, recitation practice, and comprehension exercises. They were not allowed to provide systematic pause-boundary marking, faded pause reading, pause-focused feedback, or reflection on pause placement.

**Example of PBPR prompts**

Original line:

两个黄鹂鸣翠柳，一行白鹭上青天。

Pause-marked version:

两个黄鹂 / 鸣翠柳，一行白鹭 / 上青天。

RA prompt examples:

1. Where does one meaning unit end and another begin?
2. Would the meaning be clearer if you paused here?
3. Did you pause inside a word or phrase?
4. Can you read this line again without breaking the meaning unit?
5. How did the pause help you understand the image in the poem?

**Example of MBI Prompts**

Original line:

两个黄鹂鸣翠柳，一行白鹭上青天。

MBI prompts:

1. What image is described in this line?
2. Which words show the color or movement of the scene?
3. What does the phrase “上青天” mean?
4. How are the two images related?
5. What feeling does this couplet create?

Materials Note

All poems used in the intervention were seven-character Classical Chinese poems. Different sets of poems, matched on the characteristics described in the Prosodic Reading section, were used for the pretest and posttest prosodic reading assessments. These poems were not taught during the intervention. In contrast, the CCP comprehension and classical Chinese receptive vocabulary assessments used identical items at both testing occasions. A panel of Chinese language teachers and Classical Chinese poetry experts (N= 5) evaluated all poems for difficulty, familiarity, syntactic complexity, imagery density, and pause-boundary clarity before formal implementation.

Appendix II

*List of Poems*

| No | Chinese Title | Author | English Title |
| --- | --- | --- | --- |
| 1 | 《淮上与友人别》 | 郑谷（唐） | Parting with a Friend on Huai River |
| 2 | 《渡湘江》 | 杜审言（唐） | Crossing Xiangjiang River |
| 3 | 《山亭夏日》 | 高骈（唐） | A Summer Day in Mountain Pavilion |
| 4 | 《闺怨》 | 王昌龄（唐） | A Lady’s Grief |
| 5 | 《遣怀》 | 杜牧（唐） | Recalling Past Days |
| 6 | 《江楼夕望招客》（节选七绝版） | 白居易（唐） | Inviting Friends at Riverside Tower |
| 7 | 《秋夕》 | 杜牧（唐） | An Autumn Night |
| 8 | 《齐安郡中偶题》 | 杜牧（唐） | Impromptu Verse at Qi’an Prefecture |
| 9 | 《柳州二月榕叶落尽偶题》 | 柳宗元（唐） | Fallen Banyan Leaves in Liuzhou |
| 10 | 《塞下曲・其三》 | 许浑（唐） | Frontier Ballad III |
| 11 | 《兰溪棹歌》 | 戴叔伦（唐） | Ballad of Lanxi Stream |
| 12 | 《城东早春》 | 杨巨源（唐） | Early Spring East of Town |
| 13 | 《南园・其五》 | 李贺（唐） | Southern Garden No.5 |
| 14 | 《暮春归故山草堂》 | 钱起（唐） | Returning to Old Cottage in Late Spring |
| 15 | 《题金陵渡》 | 张祜（唐） | At Jinling Ferry |
| 16 | 《晚春》 | 韩愈（唐） | Late Spring |

Appendix III

*Condition-Specific Implementation Checklist for Research Assistant Practice Teaching*

Each research assistant completed an 8–10 min practice teaching task using the same practice poem. The practice poem was not used in the formal intervention, pretest, or posttest. Each item was scored on a 3-point scale: 0 = not implemented or incorrect, 1 = partially implemented, and 2 = fully implemented. Research assistants were required to achieve at least 34 out of 40 and show no critical violation before delivering the formal intervention.

Appendix III Table A

*PBPR Group Checklist*

| Domain | Item | Score |
| --- | --- | --- |
| General preparation | 1. Introduces the poem briefly without providing extended literary interpretation. | 0 / 1 /2 |
| General preparation | 2. Explains only necessary pronunciation and difficult words. | 0 / 1 /2 |
| Condition-specific instruction | 3. Clearly explains that the lesson focuses on meaningful pauses during oral reading. | 0 / 1 /2 |
| Condition-specific instruction | 4. Defines a meaningful pause as a short break placed at a syntactic or semantic boundary. | 0 / 1 /2 |
| Condition-specific instruction | 5. Models how to identify pause boundaries based on meaning units. | 0 / 1 /2 |
| Condition-specific instruction | 6. Demonstrates at least one appropriate pause boundary using the practice poem. | 0 / 1 /2 |
| Student practice | 7. Asks students to mark pause boundaries using slashes. | 0 / 1 /2 |
| Student practice | 8. Guides students to explain why they placed a pause at a specific location. | 0 / 1 /2 |
| Student practice | 9. Conducts oral reading with full pause marks. | 0 / 1 /2 |
| Student practice | 10. Conducts oral reading with partially faded pause marks. | 0 / 1 /2 |
| Student practice | 11. Conducts independent oral reading without pause marks. | 0 / 1 /2 |
| Feedback | 12. Provides feedback on whether pauses occur at meaningful boundaries. | 0 / 1 /2 |
| Feedback | 13. Corrects inappropriate pauses within words or meaning units. | 0 / 1 /2 |
| Feedback | 14. Encourages students to avoid word-by-word reading. | 0 / 1 /2 |
| Feedback | 15. Links pause placement to meaning segmentation. | 0 / 1 /2 |
| Prohibited techniques | 16. Does not provide explicit instruction on stress or emphasis. | 0 / 1 /2 |
| Prohibited techniques | 17. Does not provide explicit instruction on intonation, emotional expression, or performance-based recitation. | 0 / 1 /2 |
| Prohibited techniques | 18. Does not conduct extended meaning-based interpretation beyond what is necessary for pause placement. | 0 / 1 /2 |
| Classroom management | 19. Maintains appropriate pacing and completes the required steps within the allotted time. | 0 / 1 /2 |
| Classroom management | 20. Uses clear, age-appropriate language suitable for Grade 7 students with reading difficulties. | 0 / 1 /2 |

*Note***.** the maximum score was 40, only those RA scored over 34 were regarded as qualified educator.

Appendix III Table B

*MBI Group Checklist*

| Domain | Item | Score |
| --- | --- | --- |
| General preparation | 1. Introduces the poem briefly and clearly. | 0 / 1 / 2 |
| General preparation | 2. Explains necessary pronunciation and difficult words. | 0 / 1 / 2 |
| Condition-specific instruction | 3. Clearly explains that the lesson focuses on understanding the meaning of the poem. | 0 / 1 / 2 |
| Condition-specific instruction | 4. Guides students to identify important words or expressions in the poem. | 0 / 1 / 2 |
| Condition-specific instruction | 5. Guides students to explain the literal meaning of selected lines. | 0 / 1 / 2 |
| Condition-specific instruction | 6. Guides students to identify imagery in the poem. | 0 / 1 / 2 |
| Condition-specific instruction | 7. Guides students to infer the emotion or theme of the poem. | 0 / 1 / 2 |
| Student practice | 8. Asks students to mark key words, imagery words, or emotional words. | 0 / 1 / 2 |
| Student practice | 9. Asks students to explain why a selected word or image is important. | 0 / 1 / 2 |
| Student practice | 10. Provides students with opportunities to discuss poem meaning. | 0 / 1 / 2 |
| Student practice | 11. Includes oral reading practice comparable to the other conditions. | 0 / 1 / 2 |
| Feedback | 12. Provides feedback on vocabulary meaning. | 0 / 1 / 2 |
| Feedback | 13. Provides feedback on sentence interpretation. | 0 / 1 / 2 |
| Feedback | 14. Provides feedback on imagery or emotional interpretation. | 0 / 1 / 2 |
| Feedback | 15. Encourages students to connect different lines into a coherent interpretation. | 0 / 1 / 2 |
| Prohibited techniques | 16. Does not ask students to mark pause boundaries. | 0 / 1 / 2 |
| Prohibited techniques | 17. Does not explicitly tell students where to pause. | 0 / 1 / 2 |
| Prohibited techniques | 18. Does not correct students’ oral reading based on pause placement. | 0 / 1 / 2 |
| Classroom management | 19. Maintains appropriate pacing and completes the required steps within the allotted time. | 0 / 1 / 2 |
| Classroom management | 20. Uses clear, age-appropriate language suitable for Grade 7 students with reading difficulties. | 0 / 1 / 2 |

*Note***.** the maximum score was 40, only those RA scored over 34 were regarded as qualified educator.

Appendix III Table C

*Control Group Checklist*

| Domain | Item | Score |
| --- | --- | --- |
| General preparation | 1. Introduces the poem in a conventional classroom style. | 0 / 1 / 2 |
| General preparation | 2. Provides a brief model reading or asks students to read aloud. | 0 / 1 / 2 |
| Standard-practice instruction | 3. Explains difficult words or expressions. | 0 / 1 / 2 |
| Standard-practice instruction | 4. Provides sentence-level translation or paraphrase. | 0 / 1 / 2 |
| Standard-practice instruction | 5. Gives a brief explanation of the poem’s background or theme. | 0 / 1 / 2 |
| Standard-practice instruction | 6. Includes regular oral reading or recitation practice. | 0 / 1 / 2 |
| Standard-practice instruction | 7. Includes conventional comprehension exercises. | 0 / 1 / 2 |
| Student practice | 8. Asks students to answer basic comprehension questions. | 0 / 1 / 2 |
| Student practice | 9. Asks students to read or recite the poem in a regular manner. | 0 / 1 / 2 |
| Student practice | 10. Provides students with opportunities to complete conventional practice tasks. | 0 / 1 / 2 |
| Feedback | 11. Provides feedback on pronunciation, word meaning, translation, or basic comprehension. | 0 / 1 / 2 |
| Feedback | 12. Keeps feedback general and consistent with standard classroom practice. | 0 / 1 / 2 |
| Treatment differentiation | 13. Does not provide systematic pause-boundary instruction. | 0 / 1 / 2 |
| Treatment differentiation | 14. Does not ask students to mark pause boundaries. | 0 / 1 / 2 |
| Treatment differentiation | 15. Does not use full pause marks, faded pause marks, and independent pause reading as a sequence. | 0 / 1 / 2 |
| Treatment differentiation | 16. Does not ask students to explain why they paused at a particular location. | 0 / 1 / 2 |
| Treatment differentiation | 17. Does not use structured meaning-based prompts beyond conventional explanation and questioning. | 0 / 1 / 2 |
| Treatment differentiation | 18. Does not include systematic meaning-unit scaffolding or written meaning reflection. | 0 / 1 / 2 |
| Classroom management | 19. Maintains appropriate pacing and completes the required steps within the allotted time. | 0 / 1 / 2 |
| Classroom management | 20. Uses clear, age-appropriate language suitable for Grade 7 students with reading difficulties. | 0 / 1 / 2 |

*Note*. the maximum score was 40, only those RA scored over 34 were regarded as qualified educator.
